# Mechanistic insights into the peroxisome proliferator-activated receptor alpha as a transcriptional suppressor

**DOI:** 10.3389/fmed.2022.1060244

**Published:** 2022-11-25

**Authors:** Tomoki Yagai, Takahisa Nakamura

**Affiliations:** ^1^Department of Metabolic Bioregulation, Institute of Development, Aging and Cancer, Tohoku University, Sendai, Japan; ^2^Division of Endocrinology, Cincinnati Children’s Hospital Medical Center, Cincinnati, OH, United States; ^3^Division of Developmental Biology, Cincinnati Children’s Hospital Medical Center, Cincinnati, OH, United States; ^4^Department of Pediatrics, University of Cincinnati College of Medicine, Cincinnati, OH, United States

**Keywords:** NAFLD, NASH, PPARα, transcriptional suppressor, transcriptional suppression

## Abstract

Non-alcoholic fatty liver disease (NAFLD) is one of the most prevalent hepatic disorders that 20-30% of the world population suffers from. The feature of NAFLD is excess lipid accumulation in the liver, exacerbating multiple metabolic syndromes such as hyperlipidemia, hypercholesterolemia, hypertension, and type 2 diabetes. Approximately 20-30% of NAFLD cases progress to more severe chronic hepatitis, known as non-alcoholic steatohepatitis (NASH), showing deterioration of hepatic functions and liver fibrosis followed by cirrhosis and cancer. Previous studies uncovered that several metabolic regulators had roles in disease progression as key factors. Peroxisome proliferator-activated receptor alpha (PPARα) has been identified as one of the main players in hepatic lipid homeostasis. PPARα is abundantly expressed in hepatocytes, and is a ligand-dependent nuclear receptor belonging to the NR1C nuclear receptor subfamily, orchestrating lipid/glucose metabolism, inflammation, cell proliferation, and carcinogenesis. PPARα agonists are expected to be novel prescription drugs for NASH treatment, and some of them (e.g., Lanifibranor) are currently under clinical trials. These potential novel drugs are developed based on the knowledge of PPARα-activating target genes related to NAFLD and NASH. Intriguingly, PPARα is known to suppress the expression of subsets of target genes under agonist treatment; however, the mechanisms of PPARα-mediated gene suppression and functions of these genes are not well understood. In this review, we summarize and discuss the mechanisms of target gene repression by PPARα and the roles of repressed target genes on hepatic lipid metabolism, fibrosis and carcinogenesis related to NALFD and NASH, and provide future perspectives for PPARα pharmaceutical potentials.

## Introduction

Peroxisome proliferator-activated receptors (PPARs) are ligand-dependent nuclear receptors belonging to the NR1C nuclear receptor subfamily, involved in lipid/glucose metabolism, inflammation, cell proliferation, and carcinogenesis (1, 2). There are three PPAR isoforms, PPARα, PPARβ/δ, and PPARγ, each with different tissue distribution and expression patterns. PPARα is abundantly expressed in the energy-producing tissues such as the liver, heart, kidney, and brown adipose tissue, whereas PPARγ is mainly expressed in the adipose tissue and macrophages, and PPARβ/δ is more widely expressed compared with PPARα (3, 4). The intense interest in PPARα is driven in part to its activation by agonists that promote upregulation of target genes related to lipid catabolism, modulating microsomal, peroxisomal, and mitochondrial fatty acid oxidation, lipoprotein metabolism, triglyceride synthesis, and gluconeogenesis (5). In the liver, these target genes are significantly involved in the pathogenesis of liver steatosis, including non-alcoholic fatty liver disease (NAFLD) and non-alcoholic steatohepatitis (NASH) (6, 7). Specifically, PPARα activation may contribute to the prevention of NAFLD and NASH aggravation because the PPARα-activated target genes have roles in anti-inflammation and reduction of lipid accumulation in the liver. PPARα activation could, therefore, be a primary pharmaceutical target (8). PPARα agonists also repress target genes (9–11) but the contribution of these “repressed target genes” to NAFLD and NASH and the mechanisms involved are not well understood. In this review, we focus on the target genes repressed by PPARα and the repression mechanisms elucidated hitherto, and discuss the potential significance of PPARα as a transcriptional suppressor.

## Main

### Physiology of peroxisome proliferator-activated receptor α

Peroxisome proliferator-activated receptor α was discovered in rodents in as primarily a carcinogen-responsible peroxisome proliferator (12). Peroxisome, a membrane-bound organelle in the cytoplasm of eukaryotic cells, performs key functions in multiple metabolic pathways such as purine catabolism, fatty acid β-oxidation, and phospholipid synthesis, in addition to the conversion of reactive oxygen species (13). PPARα is abundantly expressed in tissues metabolizing fatty acids such as liver, skeletal muscle, heart, and brown adipose tissue, in addition to inflammatory immune cells such as monocytes and macrophages (14–17). In hepatocytes, PPARα regulates peroxisomal and mitochondrial β-oxidation, lipid biogenesis and transport, cholesterol and glucose metabolism, and inflammation (18). Although PPARα protein is known to localize in the nucleus regardless of the activation state (19), in some cell types such as chondrocytes (20) and differentiated human macrophages (21), PPARα can also be found in the cytoplasm. The PPARα expression is regulated in transcriptional and post-transcriptional manners. HNF4α activates PPARα transcription by binding to the response element DR-1 in the promoter region (22), whereas COUP-TFII antagonizes the HNF4α transcriptional activity by competing with the binding to DR-1 in the promoter (23). A transcription factor KLF6 induces miR-10b that inhibits PPARα protein translation (24). A recent study showed that hepatic Argonaute 2 (Ago2) inhibits PPARα expression, suggesting that Ago2-mediated microRNA processing and RNA silencing have significant roles in PPARα repression (25).

### Structure

PPARα has four structural domains, designed A/B, C, D, and E/F domains. The N-terminal A/B domain harbors a ligand-independent transcriptional activating function (AF-1). The C domain includes DNA binding domain (DBD) essential for binding to the PPAR response element (PPRE) in the target gene promoter/enhancer sites (26). The D domain is a hinge region that includes binding sites for co-repressors such as NCoR and SMRT. The E/F domains carry ligand binding domains (LBD) that harbor a relatively larger cavity of ligands compared with other nuclear receptors (17). The binding of agonists to LBD induces the conformation change, which results in the recruitment of transcriptional complexes with co-activators and subsequent transcriptional activation (27–29).

### Selective agonists

Free fatty acids (FFAs) have been identified as endogenous agonists for PPARα. The n-3 polyunsaturated fatty acids (PUFAs), such as Eicosapentaenoic acid (EPA) and Docosahexaenoic acid (DHA), in particular, have been shown to be potent agonism compared with other FFAs (30). Such endogenous agonists come from dietary nutrients when feeding or from adipose tissues during fasting. In addition to endogenous agonists, synthetic amphipathic carboxylic acids such as fibrates that are frequently used for the treatment of hyperlipidemia and hypercholesterolemia, are demonstrable PPARα selective agonists (5). The first fibrate drugs were developed during the 1960s-1980s, although PPARα was not identified as the direct molecular target at that time. Currently, several synthetic PPARα agonists developed have been used clinically and experimentally (e.g., Fenofibrate, Clofibrate, Bezafibrate, Gemfibrozil, Pemafibrate, Wy-14,643, GW9578, GW7647) (5).

### Transcriptional gene activation by peroxisome proliferator-activated receptor α

Studies over the past decades elucidated gene transcription mechanisms of PPARα (2, 5, 31–33). Retinoid X receptor alpha (RXRα), a nuclear receptor belonging to the NR2B subfamily, is an obligate heterodimer partner for PPARα. RXR family consists of three distinct members, known as RXRα, RXRβ, and RXRγ (34, 35). RXRα (NR2B1) was the first identified RXR that is abundantly expressed in the liver. While RXRα is activated by the endogenous agonist 9-*cis* retinoid acid, ligand-independent RXRα forms a transcription complex with PPARα for the transcriptional activation. One of the significant physiological roles of PPARα is as a transcription factor activating target gene expressions (36–39). When a selective agonist binds to the LBD of PPARα, PPARα can bind to PPRE sites via heterodimerization with RXRα. PPRE sites consist of direct repeat type 1 (DR-1), which is a tandem repeat of recognition motif 5′-AGGTCA-3′ separated by a single nucleotide (40). PPARα binds to the 5′ extended half-site of the response element, whereas RXRα binds to the 3′ half-site (41, 42). Although PPARα can bind to PPRE without agonists, the interaction is not stable because of the chromatin condensed state (27–29). Furthermore, the lack of an agonist inhibits transcriptional activity of PPARα as co-repressors, such as NCOR1 and SMRT, are bound (43). Binding of the agonists PPARα releases bound co-repressors as PPARα conformation changes, and with co-activator recruitment of components, such as CBP1/P300, SRC-1, and PGC1, target gene transcription is activated (44). The binding of co-activator CBP1 is known to induce HAT activity, resulting in chromatin remodeling, which opens condensed genomic DNA to exposed PPRE sites and allows access of PPARα/RXRα heterodimer to the PPRE tightly (45). Taken together, a canonical function of PPARα is to induce target gene transcription by forming the transcription complex with RXRα and other transcriptional co-activators when the agonist binds and modifies the conformation of PPARα.

### Peroxisome proliferator-activated receptor α-mediated transcriptional gene repression

In addition to transcriptional activation, transcriptomic studies in the PPARα-activated cells and tissues indicate that there are numerous target genes whose expression is repressed by PPARα, and these repressed genes have significant roles in various homeostasis (10), although the molecular mechanisms of how PPARα suppresses target genes remain poorly studied. Gene repression mechanisms have been reported for other nuclear receptors, such as the Thyroid receptor and Glucocorticoid receptor (46), from which it is deduced that the repression mechanisms can be classified as *Trans*-acting, *Cis*-acting, or indirect manner.

### *Trans*-acting repression

**FIGURE 1:**
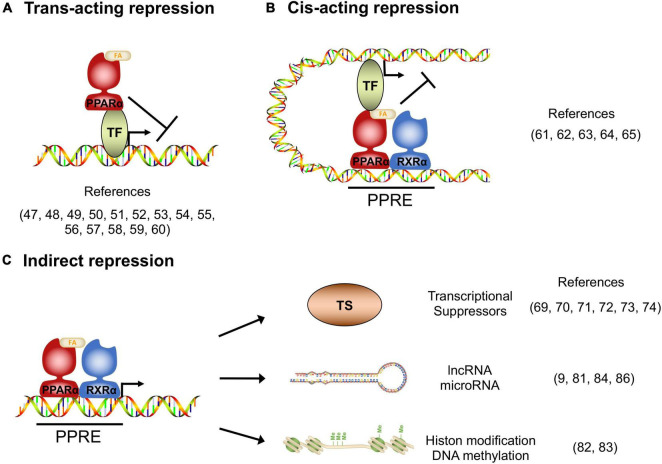
Transcriptional repression manners by PPARα. **(A)** *Trans*-acting repression manner by direct interaction between PPARα and the target transcription factors (TF). **(B)** *Cis*-acting repression manner depending on PPARα binding to the PPAR responsive element (PPRE). **(C)** Indirect repression manner, upregulating transcriptional suppressors, long non-coding RNAs, microRNAs and epigenetic modulators.

Several studies reported that the activated PPARα directly binds to transcription factors and interferes the transcriptional activity. The protein-protein interaction-mediated transcription repression is known as trans-acting repression (Figure 1A). It has been elucidated that PPARα has roles in the repression of hepatic inflammation by inhibiting Activator protein 1 (AP-1) and Nuclear factor-κB (NF-κB) pathways through trans-repression. Ligand-activated PPARα directly binds to a NF-κB component p65 and the N-terminus JNK-responsive part of c-Jun, resulting in the prevention of the unique response element binding of NF-κB and AP-1 (47). Bougarne et al. uncovered that the PPARα interference of p65 is synergistically induced with trans-repression by GR binding to p50 (48). Another study involving mice carrying mutant PPARα lacking the DBD region showed significant suppression of chronic liver inflammation by NF-κB and AP-1 pathway, suggesting that NF-κB and AP-1 suppression by PPARα is independent of the DNA binding, and that PPARα directly interacts with NF-κB and AP-1 (49). Since NF-κB and AP-1 pathways regulate the expression of pro-inflammatory cytokines, these findings suggest that PPARα may exert its anti-inflammatory effects by suppressing these pathways in the liver. Previous studies reported that saturated fatty acids activate JNK in hepatocytes (50, 51), whereas the hepatic JNK is required for AP-1 activation and NCOR1 expression (52). As NCOR1 is a potent co-repressor for PPARα, these findings suggest that the JNK-NCOR1 axis reciprocally affects the PPARα anti-inflammatory effect. In addition to NF-κB and AP-1, it was revealed that activated PPARα also binds to GRIP1/TIF2, which is a co-activator of C/EBPβ. The PPARα’s interaction with GRIP1/TIF2 results in interference of C/EBPβ binding to the response element (53). Blanquart et al. reported that the protein kinase C pathway-mediated phosphorylation of C/EBPβ at Ser179 and Ser230 residues suppresses C/EBPβ activity in the fibrinogen-β promoter (54). Oka et al. reported that PPARα and SIRT1 form a heterodimer and bind to ERR-responsive elements, leading to competitive inhibition of ERR pathway related to mitochondria respiration (55, 56). Several studies reported HNF4α inhibition by PPARα. Shin et al showed that PPARα activation decreases HNF4α protein but not mRNA, resulting in transcriptional inhibition of the HNF4α target gene ACMSD (57). Another study showed that an HNF4α target gene, Gls2, is significantly repressed by PPARα with HNF4α protein degradation (58). Recently, it was reported that PPARα/RXRα heterodimer binds to HNF4α and promotes ubiquitination, resulting in the HNF4α protein degradation and repression of the HNF4α target gene Sds promoter activity (59). Of note, Leuenberger et al reported that SUMOylated but not naïve PPARα interacts with a transcription factor GABPα and represses the transcriptional activity in Cyp7b1 promoter, indicating that PPARα is subject to protein modifications for transcriptional activity (60). Altogether, these studies suggest that direct interactions between PPARα and transcription factors may affect numerous metabolic and inflammatory pathways through gene repression.

### *Cis*-acting repression

Previous studies revealed that DNA-bound PPARα could prevent the transcription of target genes in a number of different systems. The repression manner based on protein-DNA interaction is known as *cis*-acting repression (Figure 1B). Mogilenko et al. uncovered that transcriptional activity in complement C3 promoter is inhibited by physical interactions between PPRE-bound PPARα and p65. They showed that PPARα binding to PPRE is not limited only to transcriptional activation but repression (61). ChIP-chip analysis by van der Meer et al. proposed that PPARα binding to PPRE near STAT response elements interferes with the STAT1 and STAT3 transcriptional activation in the target gene promoters such as STARD13 and TOX3 in HepG2 cells (62). You et al reported that activated PPARα binds to a PPRE located on the Glut-1 promoter, resulting in the inhibition of transcriptional activity and cancer cell proliferation (63). A recent study supported that the Glut-1 inhibition by PPARα contributes to tumor growth and chemo-resistance (64). Zhang et al showed that PPARα activation by fenofibrate recruits NCOR and associated HDAC to the INFγ gene locus, resulting in the repression of IFNγ expression in mouse T cells (65). Although the functional detail of “repressive PPRE” as a transcriptional silencer is still controversial, these studies clearly suggest that PPARα has *cis*-element-dependent gene repression mechanisms.

### Indirect repression

Several studies have reported that PPARα had mechanisms of transcriptional inhibition not only by direct interaction but also indirectly through involvement/regulation of other transcriptional regulators, long non-coding RNA (lncRNA), microRNA and epigenetic modulators (Figure 1C). Previous studies uncovered that Cyp7a1 and Cyp27a1 expression are repressed by PPARα agonism in human and rodent cells (66–68). These cytochrome P450 proteins have significant roles in bile acid synthesis, resulting in a decline in the output of bile acids and an increase in cholesterol secretion. It was reported that PPARα agonism increases a nuclear receptor Rev-erbα expression, and Rev-erbα inhibits Apoa1 gene transcription in rodents (69–71). The PPARα-activated Rev-erbα also represses Apoc3 gene expression by binding to the enhancer/promoter region (72–74). As genetic deletion of Rev-erbα leads to hepatic steatosis in mice, PPARα-mediated Rev-erbα induction appears to exert a protective role in the development of NAFLD, at least in part, by suppressing the expression of Apoa1 and Apoc3 that control lipid transport in hepatocytes (75). Makled et al. reported that a pan-agonist for PPARα/γ (a.k.a. Saroglitazar) downregulates pro-fibrotic gene expressions such as TGF-β1 and PDGF-BB, followed by the downstream target gene repressions such as PAI-1 and Smad-3 in rat liver fibrosis model (76, 77). The results suggest that PPARα (and also PPARγ) can transcriptionally repress the TGF-β signaling pathway. Bansal et al identified that the AF-1 domain of activated PPARα directly binds to the kinase domain of TAK-1 protein and prevents phosphorylation. Phosphorylation of TAK-1 is a molecule switch of the TGF-β signal cascade, indicating that the inhibition of phosphorylation results in the prevention of the TGF-β pathway (78). Brocker et al unveiled that hepatic PPARα directly upregulates a lncRNA Gm15441. Gm15441 expression suppresses its antisense transcript encoding TXNIP, resulting in inflammasome activation, CASP1 cleavage and proinflammatory IL-1β maturation (9). These findings suggest that PPARα regulates the expression of lincRNAs relevant to the development of steatohepatitis. Although precise mechanisms are still unclear, it was reported that PPARα activation represses Oleate-inducible Fatp1 expression, attenuating total free fatty acid and triglyceride accumulation in macrophages (79). Triglyceride accumulation is related to macrophage activation (80), suggesting that the Fatp1 repression by PPARα is related to inflammation. Furthermore, a recent study unveiled that the let-7 microRNA family is significantly repressed by PPARα agonism and the let-7 microRNA prevented RXRα ubiquitination through RNF8 mRNA decay. RXRα degradation results in the inhibition of the transcriptional activity of the PPARα/RXRα complex, indicating that PPARα - let-7 microRNA - RNF8 - RXRα axis is a negative feedback loop in the hepatic lipid metabolism (81).

Several target genes repressed by PPARα are related to cancer progression and tumor growth. A study unveiled that intestinal PPARα upregulated RB1 expression in mouse colon, resulting in the repression of DNMT1 and PRMT6. DNMT1 and PRMT6 contribute to the inhibition of tumor suppressor genes such as Cdkn1a and Cdkn1b via DNA methylation and histone H3R2 dimethylation (82). Another study reported that hepatic PPARα upregulates a transcription factor E2F8 in mice. The E2F8 increases Uhrf1 expression, contributing to DNA methylation in the Cdh1 promoter and the inhibition of expression (83). CDH1 represses proto-oncogene Myc expression through the Wnt pathway, suggesting that the PPARα-CDH1 pathway may enhance tumor growth. Shah et al. uncovered that PPARα agonism repressed at least twelve microRNA expressions in mouse liver. Especially the repressed target gene let-7C microRNA targets Myc mRNA and decays the stability (84). These studies indicate that rodent PPARα has multiple roles in the promotion of carcinogenesis. It is consistent with previous publications showing that long-term activation of rodent PPARα induces carcinogenesis (5). In contrast, Shi et al suggested that PPARα activation represses E2F1 transcriptional activity and the target gene expressions via the p21 pathway, modulating transcriptional complexes of E2F1 and pRB in human glioma cells (85). In human glioma cells, another study showed that PPARα upregulated miR-214 expression, resulting in E2F2 mRNA decay and inhibition of cell proliferation (86). These studies suggest that PPARα-repressed target genes contribute to the inhibition of cancer progression and tumor growth in humans.

## Discussion

Studies about repressed target genes of PPARα are not sufficiently understood compared with those of the activated target genes. However, it has become obvious that one of the critical functions of PPARα is to exert transcriptional suppression of its target genes through multiple mechanisms (Table 1). The repressed target genes include various master regulators related to inflammation, fibrosis, and carcinogenesis, which contribute to, at least in part, the physiological roles of PPARα and the beneficial effects of PPARα agonist treatment. Given that PPARα represses the major pro-inflammatory transcriptional regulators, NF-κB and AP-1 pathways, the mechanisms of PPARα-mediated gene suppression may play a significant role in exacerbating hepatic inflammation (87). In addition, PPARα represses other transcriptional regulators/pathways, such as GRIP1/TIF2, HNF4α, IFNγ and TGF-β, that are related to lipid metabolism and inflammation, which appear to contribute to the beneficial effects of PPARα activation in hepatocytes. When PPARα is active as a transcription activator, the activated PPARα generally forms a transcriptional activation complex with co-activators, and the physical contact with the transcription factors accelerates their transcriptional activities. Conversely, the molecular mechanisms of how PPARα suppresses the target gene transcription and whether PPARα requires to form a specific transcriptional suppression complex to be a transcriptional suppressor remain to be elucidated. Although several previous studies identified PPREs located near repressed target gene promoter/enhancer, the sequential and positional differences between activating and repressing PPREs are still unclear. In addition, PPARα-mediated gene repression and activation occur at approximately the same time upon agonist treatment. As several epigenetic repression mechanisms have been shown in rodent cancer models and inflammation, the differences in epigenetic modifications in PPREs may be involved in the regulation of gene repression or activation of PPARα’s target genes. Identifications of the elements that distinguish PPRE enhancers from silencers would drastically advance our knowledge of PPARα biology. In addition, PPARα protein modifications, including SUMOylation and phosphorylation, may also impact PPARα-mediated gene repression and activation. One study demonstrated that SUMOylation of PPARα accelerates trans-acting repression (60), whereas another showed that post-translational phosphorylation of PPARα has a significant role in the trans-repression (54). Although the detail of mechanisms needs further analyses, such protein modifications could be related to the binding affinity of PPARα to the PPRE enhancer or silencer, or the other repressed target genes. At present, one PPARα agonist (a.k.a. Pemafibrate) and three pan-PPAR agonists (a.k.a. Lanifibranor, Pioglitazone, and Saroglitazar) are under clinical trials as drugs for NASH treatment respectively (88–91). Novel insights into the mechanisms will help the process of current clinical trials.

**TABLE 1:** Summary of repressed target genes of PPARα.

*Trans*-acting repression	Target TFs	Function	References
	p65 (NF-KB)	Transcription of pro-inflammatory genes	(47–49)
	c-Jun (AP-1)	Transcription of cell proliferation and apoptosis related genes	(47, 49)
	GRIP1/TIF2	Co-activator for nuclear receptors	(53)
	SIRT1	Deacetylation of transcription factors	(55, 56)
	HNF4α	Transcription of metabolism related genes	(57–59)
	GABPα	Transcription of metabolism related genes	(60)

***Cis*-acting repression**	**Target *cis*-element**	**Repressed gene expression**	**References**

	Complement C3 promoter	Complement C3	(61)
	STAT response element	STAT target genes	(62)
	Glut-1 promoter	Glut-1	(63, 64)
	IFNy gene locus	IFNy	(65)

**Indirect repression**	**Direct targets**	**Repression mechanism**	**Repressed indirect target**	**Function**	**References**

	Unknown	Unknown	Cyp7a1, Cyp27a1	Decrease of bile acid synthesis and increase of cholesterol secretion	(66–68)
	Rev-erbα	Suppression of the target transcription	Apoal, Apoc3	Modification of lipid metabolism	(69–74)
	Unknown	Unknown	TGF-β1, PDGF-BB	Repression of PAI-1, Smad-3 expressions	(76, 77)
	TAK-1	Prevention of TAK-1 phosphorylation	TGF-β target genes	Repression of TGF-β pathway	(78)
	Gm15441 IncRNA	Inhibition of antisense transcript in the locus	TXNIP	Inflammasome activation, CASP1 cleavage, IL-1 β maturation	(9)
				Attenuation of fatty acid and	
	Unknown	Unknown	Fatpl	Triglyceride accumulation in macrophage	(79)
	Unknown	Unknown	let-7 microRNA family	Promotion of RXRa ubiquitination through RNF8	(81)
	RB1	DNA methylation and histon H3R2 modification through DNMT1 and PRMT6	Cdknla, Cdknlb	Inhibition of tumor suppression	(82)
	E2F8	DNA methylation through Uhrf 1	Cdh1	Enhancement of tumor growth through myc activation	(83)
	unknown	unknown	let-7C microRNA	Enhancement of tumor growth through myc activation	(84)
	Unknown	Unknown	E2F1	Inhibition of cancer cell proliferation	(85)
	miR-214	mRNA decay and translational inhibition	E2F2	Inhibition of cancer cell proliferation	(86)

## Author contributions

Both authors listed have made a substantial, direct, and intellectual contribution to the work, and approved it for publication.
